# Supplementary material for: Paving the way for rural revitalization: Empirical analysis of the ‘Sihao Rural Road Policy’ and transport mode choice in Inner Mongolia, China
Source: PLoS One. 2025 May 19;20(5):e0324026. doi: 10.1371/journal.pone.0324026 (PMC12087995; doi:10.1371/journal.pone.0324026)
Supplement: S1 File — (PDF) [file pone.0324026.s001.pdf]

内蒙古开放大学

科研伦理审查批件/意见

编号［2023］KY 第 SSR2302 号

项目名称	内蒙古四好农村路建设对乡村经济的影响分析研究		
申请人	宝音图	申请学院/系	经济系
审查方式	□会议审查 ☑ 快速审查		
审查内容	课题/项目伦理申请审核流程，伦理审查申请表，研究方案，知情同意书		

伦理审查委审查决定

伦理审查委员会同意该研究项目通过伦理审查，允许项目实施。申请人在项目实施过程中应严格遵循伦理规范，并及时向伦理审查委员会汇报任何与伦理相关的变更或意外事件。

审查委员会签名：

签字日期：2023 年 7 月 27 日
