# Supplementary material for: Paving the way for rural revitalization: Empirical analysis of the ‘Sihao Rural Road Policy’ and transport mode choice in Inner Mongolia, China
Source: PLoS One. 2025 May 19;20(5):e0324026. doi: 10.1371/journal.pone.0324026 (PMC12087995; doi:10.1371/journal.pone.0324026)
Supplement: S2 File — (PDF) [file pone.0324026.s002.pdf]

内蒙古开放大学

ᠥᠪᠦᠷ ᠮᠣᠩᠭᠣᠯ ᠤᠨ ᠨᠡᠭᠡᠭᠡᠯᠲᠡ ᠶᠡᠬᠡ ᠰᠤᠷᠭᠠᠭᠤᠯᠢ

Research Ethics Review Approval/Opinion

No. [2023] KY SSR2302

Project Title	Research on the Impact of the 'Four Good Rural Roads' Construction on Rural Economic Development in Inner Mongolia		
Applicant	Bao Yintu	Department	Department of Economics
Review Method	☐ Committee Review ☑ Expedited Review		
Review Content	Ethics review application process, ethics review application form, research proposal, informed consent form.		

Decision of the Ethics Review Committee

The Ethics Review Committee agrees that this research project has passed the ethics review and permits the project to proceed. The applicant must strictly adhere to ethical guidelines during the implementation of the project and promptly report any changes or unforeseen events related to ethics to the Ethics Review Committee.

Signature of the Review Committee:

Date of Signature: July 27, 2023
