# Supplementary material for: Paving the way for rural revitalization: Empirical analysis of the ‘Sihao Rural Road Policy’ and transport mode choice in Inner Mongolia, China
Source: PLoS One. 2025 May 19;20(5):e0324026. doi: 10.1371/journal.pone.0324026 (PMC12087995; doi:10.1371/journal.pone.0324026)
Supplement: Attachment — Submitted filename: Response to Reviewers.docx [file pone.0324026.s006.docx]

**Professor Hironori Kato (Academic Editor)**

***PLOS ONE***

**February 18, 2025**

**Manuscript ID:** PONE-D-24-41259

**Title:** Paving the Way for Rural Revitalization: Empirical Analysis of the ‘Sihao Rural Road Policy’ and Transport Mode Choice in Inner Mongolia, China

**Final Revision Deadline:** February 20, 2025

**Dear Prof. Hironori Kato:**

Thanks a lot for your kind email on January 07, 2024, regarding our manuscript entitled "*Paving the Way for Rural Revitalization: Empirical Analysis of the ‘Sihao Rural Road Policy’ and Transport Mode Choice in Inner Mongolia, China*" and all the professional and constructive comments from two devoted reviewers. We are profoundly grateful to you and two reviewers for evaluating our manuscript comprehensively by dedicating your valuable time and effort to provide us with your beneficial suggestions/comments.

We have thoroughly considered all the requirements from two contributed reviewers, and we have made substantial amendments to the initial manuscript based on two reviewers' recommendations. We attach our revised manuscript documents with the tracked changes to highlight the revisions and a point-by-point response to two dedicated reviewers' constructive and specialized comments. We have also addressed the additional journal requirements outlined in the decision letter and have ensured compliance with the revision deadline.

The revised manuscript is a suitable response to the comments and significantly improves the initial submission (manuscript). We expect it is now eligible for being further considered and reviewed again by *PLOS ONE*. If there are any problems we missed, please do not hesitate to contact us and we will try our best effort to respond to them.

Finally, we should show our deep appreciation again in advance of our revised manuscript for your thoughtful consideration. Please retake your valuable time to review the modifications we have made insight. Thank you for your kind contribution again. Wish you have a wonderful day.

Sincerely yours,

Dr. Ligao Bao

Tokyo University of Agriculture and Technology

Graduate School of Engineering Tokyo University of Agriculture and Technology, 2-24-16, Naka-cho, Koganei-shi, Tokyo, 184-8588, Japan

Email: bolagtuat@gmail.com

ORCID: 0000-0003-3579-1845

**Reviewers' comments:**

**Reviewer's Responses to Questions**

**Comments to the Author**

**1. Is the manuscript technically sound, and do the data support the conclusions?  The manuscript must describe a technically sound piece of scientific research with data that supports the conclusions. Experiments must have been conducted rigorously, with appropriate controls, replication, and sample sizes. The conclusions must be drawn appropriately based on the data presented.**

**Reviewer #1: Partly**

**Reviewer #2: Partly**

**Response:** We acknowledge the reviewers’ concerns and have carefully revised the manuscript to ensure that the analysis aligns with the conclusions. We have refined the discussion, improved the interpretation of findings, and added robustness checks, including village fixed effects, to strengthen the validity of the results.

**2. Has the statistical analysis been performed appropriately and rigorously?**

**Reviewer #1: No**

**Reviewer #2: No**

**Response:** We have revised our statistical approach to enhance its rigor. Specifically, we have:

•	Included village fixed effects to account for potential unobserved heterogeneity.

•	Clarified the model specification and addressed any inconsistencies in the interpretation of direct and indirect effects.

•	Improved the presentation of statistical results, ensuring clarity and transparency in model estimations.

**3. Have the authors made all data underlying the findings in their manuscript fully available?**

**The PLOS Data policy requires authors to make all data underlying the findings described in their manuscript fully available without restriction, with rare exception (please refer to the Data Availability Statement in the manuscript PDF file). The data should be provided as part of the manuscript or its supporting information, or deposited to a public repository. For example, in addition to summary statistics, the data points behind means, medians and variance measures should be available. If there are restrictions on publicly sharing data—e.g. participant privacy or use of data from a third party—those must be specified.**

**Reviewer #1: Yes**

**Reviewer #2: No**

**Response:** We acknowledge Reviewer #2’s concern regarding data availability. The dataset used in this study contains information that cannot be publicly shared due to privacy and confidentiality constraints. However, the data will be made available upon reader’s reasonable request to the corresponding author, in line with ethical and institutional guidelines.

**5. Review Comments to the Author**

**Please use the space provided to explain your answers to the questions above. You may also include additional comments for the author, including concerns about dual publication, research ethics, or publication ethics. (Please upload your review as an attachment if it exceeds 20,000 characters)**

**Reviewer #1: - Thank you very much for giving us the opportunity to review this interesting paper. The paper’s value is to assess the effects of a rural road program on people’s travel behaviors after rural road improvement. The following could be addressed to strengthen the discussions in the paper and help readers’ understanding.**

**Response:** Thank you for your encouraging comments and for recognizing the contribution of our manuscript. We appreciate your careful reading. Below, we address your specific suggestion in detail:

**- Please ensure that the paper should be proofread carefully during revision for second review.**

**Response:** We have carefully proofread the manuscript and made several revisions to improve its clarity, readability, and academic tone. Specific grammatical, structural, and typographical errors have been corrected to enhance the overall quality of the paper.

**- 2.1. Please clarify why this paper needs to focus on travel behaviors when you measure the impact of “Sihao Rural Road.” This explanation is needed to justify your 3 key questions in Section 4.1.**

**Response:** To improve the logical flow and strengthen the justification for analyzing travel behavior, we have moved the three key research questions from Section 4.1 (Line:401-413) to the Introduction section. (Line:121-138)

We utilize an integrated Structural Equation Modeling (SEM) and Discrete Choice Modeling (DCM) framework to capture both the direct and indirect effects of the SRRP on travel behavior, thus offering a more nuanced understanding of the mechanisms behind these changes.

In particular, the research addresses the following questions:

(1) Does the SRRP continue to exert significant direct effects on car ownership, travel frequency, travel distance, and travel mode choice after controlling for socio-demographic factors?
(2) How does the SRRP indirectly influence travel mode choice through its effects on mediating variables such as car ownership, travel frequency, and travel distance?
(3) What is the overall impact of the SRRP on travel mode choice, combining both direct and indirect effects?

By examining these questions, the study aims to provide a comprehensive perspective on how rural infrastructure improvements like the SRRP shape travel behavior. Findings from this research can inform policy recommendations for future rural road development and identify ways to optimize infrastructure investments to achieve broader socio-economic benefits and sustainable mobility patterns.

Additionally, we have explicitly clarified why measuring the impact of the Sihao Rural Road (SHRR) program necessitates a focus on travel behaviors. (Line:88-115)

The decision to focus on travel behavior stems from the critical role that daily mobility plays in realizing the broader benefits of rural road improvements. Enhanced rural roads can fundamentally reshape how often residents travel, how far they go, and which modes of transport they prefer. These changes in travel behavior mediate a wide range of socioeconomic outcomes: improved roads may reduce the time and financial costs of commuting, prompt shifts in mode preference (e.g., from walking to motorized modes), and influence household decisions about vehicle ownership. By capturing these direct and indirect pathways, researchers gain a more comprehensive understanding of how the SRRP translates into real-world changes in mobility patterns—ultimately affecting economic opportunities, access to services, and social inclusion.

The SRRP, initiated in 2015, is particularly interesting because it aims to fix the differences in transport infrastructure between urban and rural areas (MOT, 2015). By focusing on improving the quality and connectivity of rural roads, the policy aspires not only to make travel more convenient but also to foster new economic opportunities in rural areas. This could help close the persistent urban–rural income gap, a central concern for sustainable development. Through enhanced transport links, rural communities could see boosts in employment, market access, and overall well-being (MOT, 2019). Despite the program’s potential, there is still limited empirical evidence demonstrating how specific aspects of the SRRP—like upgraded road infrastructure and enhanced road management—translate into shifts in travel behavior and ultimately contribute to rural revitalization.

This revision ensures a stronger connection between our research objectives and the empirical analysis.

**- Please include the potential effects of the relatively small sample size (127 households) on the overall modeling results, probably in Section 5. Furthermore, please provide challenges you faced during the data collection, which resulted in the removal of 73 sample households.**

**Response:** We appreciate the reviewer’s concern about the sample size and have added the following discussion to Section 3.3 and 5:

1. Data Collection Challenges: (Line:291-296)

We used a stratified sampling technique to select 200 households for the survey, which was distributed across four villages village A, village B, village C, and village D in Qingshuihe County. However, due to incomplete questionnaires (n = 10), data inconsistencies (n = 18), and non-responses (n = 45), a total of 73 responses were excluded. As a result, the final sample for analysis comprised 127 valid responses.

2. Sample Size Limitations:

While the final sample of 127 households provided sufficient statistical power to detect medium-to-large effect sizes within the structural equation modeling (SEM) framework, it may have limited the detection of smaller interaction effects. To acknowledge this limitation, we have added a discussion in Section 5, emphasizing the need for future studies with larger and more diverse samples to enhance the robustness of the findings. (Line:634-638)

Despite the valuable insights provided by this study, several limitations should be acknowledged. First, the sample size is relatively small (127 households across four villages), which may limit the generalizability of the findings to other rural areas in China or beyond. While the inclusion of village fixed effects improves robustness, additional research with a larger and more diverse sample could help validate and refine the observed relationships.

**- 3.3. Please describe briefly each village's socio-economic characteristics and road density. In the modeling exercise, village-level variables were not included. Villages might be influential factors to people's livelihoods and travel behavioral patterns, however, the study villages' differences are not detailed other than whether the Sihao Rural Road program has been applied or not.**

**Response:** Thank you for your suggestion, to address this concern, we have added a detailed description of the socio-economic characteristics and road density of each study village in Section 3.3 (The study area). (Line:303-321)

Table 1 presents key characteristics for the four study villages. Village A shows a moderate road density (1.15 km/km²), lower average income (14,500 yuan), and high reliance on agriculture (81%), suggesting a stronger dependence on traditional, local transport modes. Village B, which did not implement the SHRR, has a low road density (0.71 km/km²) but a high paved road percentage (93%) and an average income of 16,500 yuan—indicating that prior infrastructure investments have improved road quality, even if internal connectivity remains limited. In contrast, Village C stands out with the highest road density (2.3 km/km²), the lowest transport cost (5 yuan) and travel time (5 minutes) to the county center, an average income of 15,000 yuan, and a lower agricultural labor share (65%), reflecting superior connectivity and accessibility. Village D, which implemented the SHRR, shares a low road density (0.71 km/km²) with Village B but has the highest average income (17,000 yuan) and a moderate agricultural labor share (80%), along with higher transport costs (15 yuan) and longer travel time (20 minutes). For broader context, the average income in Qingshuihe County (24,110 yuan) is notably higher than that of the study villages, indicating economic disparities between rural and county-level populations. Moreover, the county’s overall road density (0.65 km/km²) is lower than that of all study villages, as much of the county consists of non-residential land such as mountains, forests, and agricultural fields. This suggests that while rural areas face infrastructural challenges, localized road investments, including the SHRR program, may have enhanced connectivity in specific villages.

| Table 1 | | | | | | | |
| --- | --- | --- | --- | --- | --- | --- | --- |
| Village characteristics. | | | | | | | |
|  | Village A | Village B | Village C | Village D | Qingshuihe County | |  |
| Village Area (km²) | 20 | 35 | 8.7 | 35 | | 2818 |  |
| Road Density (km/km²) | 1.15 | 0.71 | 2.3 | 0.71 | | 0.65 |  |
| Paved Road Percentage (%) | 85 | 93 | 90 | 85 | | N/A |  |
| Average Transport Cost (Yuan) | 10 | 15 | 5 | 15 | | N/A |  |
| SHRR | Yes | No | No | Yes | | N/A |  |
| Road Upgrades in Last 5 Years (km) | 2 | 16 | 2 | 10 | | 781 |  |
| Avg Travel Time to County Center (min) | 15 | 25 | 5 | 20 | | N/A |  |
| Average income (Yuan) | 14500 | 16500 | 15000 | 17000 | | 24110 |  |
| Population (People) | 434 | 998 | 1285 | 1742 | | 136400 |  |
| Ratio of Labor Force Engaged in Agriculture (%) | 81 | 80 | 65 | 80 | | N/A |  |

Source: Survey data and the Statistical Yearbook.

**- 4.1.**

**a. Please add the discussion on whether any other government programs or policies or any infrastructure development projects were implemented in parallel with the Sihao Rural Road program. If any of the programs/policies/projects existed, please explain how you eliminated those programs/policies’ effects on the variables for this paper, particularly income level, agricultural sectors, health conditions, education, and travel modal ownership.**

**Response:** We appreciate your suggestion and acknowledge that infrastructure improvements and policy interventions do not occur in isolation. To address this concern, we have incorporated a discussion in Section 3.3 regarding other government programs and policies that were implemented alongside the SHRR program. (Line:323-335)

In the study area of Qingshuihe County, government-led initiatives such as targeted poverty alleviation, healthcare subsidies, and improvements to local schools and agricultural facilities have been implemented in recent years alongside the SHRR Program. To minimize potential confounding effects from these parallel policies, we employed two approaches. First, the sample villages were deliberately selected to include both households benefiting from SHRR improvements and those that did not, ensuring relative uniformity in exposure to other initiatives. Informal interviews with local officials and community leaders further confirmed that no major road-related or large-scale development programs were introduced during the study period that could disproportionately affect travel behavior. Second, the household survey included questions on recent policy-driven changes in income, agricultural activities, health services, education access, and vehicle ownership, enabling us to detect any unusual shifts unrelated to SHRR. These measures help ensure that our analysis more accurately isolates the impact of the SHRR program from other local influences.

Furthermore, we introduced Section 4.5: Robustness Check to validate our findings by integrating village-level fixed effects into the model estimation. This additional analysis helps isolate the impact of the SHRR program by controlling for unmeasured village-specific characteristics that may influence car ownership, travel behavior, and mode choice.   (Line:565-597)

**4.5. Robustness check**

The primary analysis identified a significant relationship between the SHRR program and travel behavior. However, the interpretation of these results could be influenced by unobserved heterogeneity across villages. Since the study area consists of four distinct villages, each with unique socio-economic and infrastructural characteristics, there is a possibility that village-specific factors—such as local economic conditions, alternative transportation availability, land use patterns, or cultural attitudes toward mobility—may affect travel behavior independently of SHRR implementation.

To address these concerns, we re-estimated our models by introducing village fixed effects, using a dummy variable approach where three villages were included as categorical controls, with one village serving as the reference category. The results, presented in Table 6 and 7, confirm the robustness of our initial findings while highlighting some variations in the magnitude of estimated effects.

Table 6 presents the standardized direct, indirect, and total effects of key variables on car ownership, travel frequency, and travel distance while controlling for village fixed effects. The results remain consistent with our initial estimation, reinforcing the negative impact of SHRR on car ownership (direct effect = -0.37, p < 0.05). The coefficient for SHRR on travel frequency remains strongly negative (direct effect =-4.638, p < 0.01; total effect == -4.319, p < 0.01) and exhibits a significant positive effect on travel distance (direct effect =1.202, p < 0.001; total effect = 1.011, p < 0.01). The inclusion of village fixed effects does not alter our finding.

| **Table 6** | | | | | | | | | |
| --- | --- | --- | --- | --- | --- | --- | --- | --- | --- |
| Standardized direct, indirect and total effects on car ownership, travel frequency and travel distance with village fixed effect. | | | | | | | | | |
| Variables | Car_ownership | Average_week_times_goout | | | Average_travel_distance | | |  |  |
|  | Direct | Direct | Indirect | Total | Direct | Indirect | Total | |  |
|  | effect | effect | effect | effect | effect | effect | effect | |  |
| Car_ownership |  | -0.863 |  | -0.863 | 0.516** |  | 0.516** | |  |
|  |  | (-1.19) |  | (-1.19) | (2.90) |  | (2.90) | |  |
| Age | -0.013* | -0.015 | 0.011 | 0.004 | 0.009 | -0.007 | -0.002 | |  |
|  | (-2.05) | (-0.29) | (1.03) | (-0.08) | (0.72) | (-1.67) | (0.20) | |  |
| Education | -0.045 | 0.44 | 0.039 | 0.479 | -0.088 | -0.023 | -0.111 | |  |
|  | (-0.92) | (1.11) | (0.73) | (1.21) | (-0.90) | (-0.88) | (-1.11) | |  |
| Self_health | -0.121 | 0.41 | 0.105 | 0.514 | -0.021 | -0.062 | -0.084 | |  |
|  | (-1.62) | (0.67) | (0.96) | (0.85) | (-0.14) | (-1.41) | (-0.55) | |  |
| Agri_work_share | -0.086 | 0.858 | 0.074 | 0.933 | -0.108 | -0.044 | -0.153 | |  |
|  | (-0.41) | (0.50) | (0.38 | (0.54) | (-0.26) | (-0.40) | (-0.35) | |  |
| Incomelevel_22 | -0.12* | 0.207 | 0.104 | 0.311 | -0.007 | -0.062 | -0.069 | |  |
|  | (-2.40) | (0.50) | (1.07) | (0.77) | (-0.07) | (-1.85) | (-0.67) | |  |
| BK | -0.032 | -0.071 | 0.027 | -0.044 | 0.098 | -0.016 | 0.082 | |  |
|  | (-0.52) | (-0.15) | (0.48) | (-0.09) | (0.82) | (-0.51) | (0.66) | |  |
| SHRR | -0.37* | -4.638** | 0.3196 | -4.319** | 1.202*** | -0.191 | 1.011** | |  |
|  | (-2.09) | (-3.18) | (1.04) | (-2.99) | (3.35) | (-1.69) | (2.77) | |  |
| Village fixed effect | YES | YES | YES | YES | YES | YES |  | |  |
| *R^2^* | 0.127 | 0.201 |  |  | 0.291 |  |  | |  |

Note: 1) *t* statistics in parentheses 2) * p<0.05, ** p<0.01, *** p<0.001

Table 7 presents the results from the integrated SEM and DCM estimation of travel mode choice with village fixed effects. The key relationships observed in the initial model remain robust, confirming that SHRR exerts a significant positive effect on electric bicycle adoption (direct effect = 4.298, p < 0.01; total effect = 3.616, p < 0.01). For motorized modes, SHRR also demonstrates a strong direct effect (3.252, p < 0.01) and total effect (2.567, p < 0.05), reinforcing the argument that enhanced rural roads encourage the use of electric bicycle and motorized mode. The shift in statistical significance for the total effect of SHRR on motorized mode adoption highlights the necessity of accounting for village-specific characteristics when analyzing rural mobility patterns. In summary, the robustness check with village fixed effects confirms that SHRR significantly influences travel mode choices, promoting both electric bicycle and motorized mode adoption.

| **Table 7** | | | | | | | |
| --- | --- | --- | --- | --- | --- | --- | --- |
| Integrated SEM and DCM model estimation for standardized direct, indirect and total effects on travel mode choice with village fixed effect. | | | | | | | |
| Variables | Electric bicycle | | | Motorized mode | |  |  |
|  | Direct | Indirect | Total | Direct | Indirect | Total |  |
|  | effect | Effect | effect | effect | effect | effect |  |
| Car_ownership | 1.46 | -0.231 | 1.229 | 2.235*** | -0.131 | 2.103*** |  |
|  | (1.84) | (-0.94) | (1.59) | (3.40) | (-0.63) | (3.35) |  |
| SHRR | 4.298** | -0.682 | 3.616** | 3.252** | -0.685 | 2.567* |  |
|  | (3.05) | (-1.21) | (2.72) | (2.80) | (-1.27) | (2.31) |  |
| Average_travel_distance | -0.41 |  | -0.41 | -0.19 |  | -0.19 |  |
|  | (-1.16) |  | (-1.16) | (-0.61) |  | (-0.61) |  |
| Average_week_times_goout | 0.006 |  | 0.006 | 0.029 |  | 0.029 |  |
|  | (0.07) |  | (0.07) | (0.38) |  | (0.38) |  |
| Household characteristics | Yes | Yes | Yes | Yes | Yes | Yes |  |
| Village fixed effect | Yes | Yes | Yes | Yes | Yes | Yes |  |
| LL_K_/LRI | -745.5922/0.8245 | | | | | |  |
| AIC/Adjust BIC | 267.687/276.22 | | | | | |  |

Note: 1) *t* statistics in parentheses 2) * p<0.05, ** p<0.01, *** p<0.001

These additions strengthen the internal validity of our results and ensure that our conclusions about the SHRR program’s effects on travel behavior are not confounded by parallel policy initiatives.

**b. The information in Table 2 does not support the descriptive explanation of the sample in Section 4.1. Please check the internal linkages. Major items are pointed out below for your consideration.**

**Response:** We have carefully reviewed and revised Table 2 (Table 3) and its accompanying discussion in Section 4.1 to ensure that the descriptive statistics align with the reported values. (Line:382-400)

| Table 3 | | | | | |  |
| --- | --- | --- | --- | --- | --- | --- |
| Descriptive statistics. | | | | | |  |
| Variables | Obs. | Mean / Percentage Share | S.D. | Min | Max | |
| TM_3 | 127 | 2.188976 | 0.8795046 | 1 | 3 | |
| Car_owners~p | 127 | 0.9685039 | 0.5900902 | 0 | 2 | |
| Average_we~t | 127 | 9.787402 | 4.903238 | 1 | 30 | |
| Average_tr~e | 127 | 2.80315 | 1.291236 | 1 | 5 | |
| SHRR | 127 | 0.5590551 | 0.4984666 | 0 | 1 | |
| Age | 127 | 55.7874 | 8.438978 | 30 | 76 | |
| Incomelev~22 | 127 | 2.787402 | 1.051302 | 1 | 9 | |
| Agri_work_~e | 127 | 0.6232283 | 0.2775771 | 0.1 | 1 | |
| Self_health | 127 | 1.850394 | 0.7671711 | 1 | 5 | |
| Education | 127 | 3.149606 | 1.241343 | 2 | 7 | |
| Primary school | 44 | 34.65 |  |  |  | |
| Middle school | 47 | 37.01 |  |  |  | |
| High school | 24 | 18.9 |  |  |  | |
| Junior college | 9 | 7.09 |  |  |  | |
| University | 3 | 2.36 |  |  |  | |
| BK | 125 | 1.72 | 0.8576036 | 1 | 4 | |
| Improvement in transportation facilities | | |  |  |  | |
|  | 57 | 45.6 |  |  |  | |
| Upgrade or purchase of vehicles | | |  |  |  | |
|  | 57 | 45.6 |  |  |  | |
| Changes in economic conditions | | | | | | |
|  | 11 | 8.8 |  |  |  | |

The mean of *Average_week_times_goout* is 9.79, reflecting a range from 1 to 30 weekly outings and suggesting varied mobility patterns. *Average_travel_distance* has a mean of 2.803, indicating that most trips fall within the 6-10 km range. The majority of respondents (32.28%) travel between 11-20 km, followed by those who travel less than 1 km (25.98%) and 6-10 km (25.20%). Only 5.51% of respondents travel more than 20 km. The *SHRR* variable reveals that slightly more than half of the villages (mean = 0.559) have implemented SHRR projects, with a relatively balanced distribution across the sample.

The household heads' mean *Age* is 55.79 years, with most being middle-aged or older, and there is moderate variation in income levels (*Incomelevel_22* mean = 2.787), suggesting that the sample is primarily composed of low- to middle-income households. *Agri_work_share* indicates a significant reliance on agriculture, with a mean of 0.623, though this dependence varies across households. Meanwhile, the mean of *Self_health* is 1.850, suggesting generally good health among respondents. *Education* data reveal that 37.01% of household heads have completed middle school, and 34.65% have a primary school education. Finally, the *BK* variable (Primary Factor Affecting Travel) shows that 45.60% of household heads identified improvement in transportation facilities as the main factor, while another 45.60% cited the upgrade or purchase of vehicles. A smaller portion, 8.80%, indicated relocation of home or workplace as the primary factor.

**c. Please add the discussion on the average and mean income and the economic dependency on the agriculture sector of the study country and each study village, as these factors are influential to people’s decision to obtain transport means, particularly motorized ones. Also, the discussion will substantiate “the sample is primarily composed of low- to middle-income households” after Table 2.**

**Response:** We have expanded the discussion in Section 3.3 to include additional details on income levels and agricultural dependency at both the county and village levels. (Line:303-321)

Table 1 presents key characteristics for the four study villages. Village A shows a moderate road density (1.15 km/km²), lower average income (14,500 yuan), and high reliance on agriculture (81%), suggesting a stronger dependence on traditional, local transport modes. Village B, which did not implement the SHRR, has a low road density (0.71 km/km²) but a high paved road percentage (93%) and an average income of 16,500 yuan—indicating that prior infrastructure investments have improved road quality, even if internal connectivity remains limited. In contrast, Village C stands out with the highest road density (2.3 km/km²), the lowest transport cost (5 yuan) and travel time (5 minutes) to the county center, an average income of 15,000 yuan, and a lower agricultural labor share (65%), reflecting superior connectivity and accessibility. Village D, which implemented the SHRR, shares a low road density (0.71 km/km²) with Village B but has the highest average income (17,000 yuan) and a moderate agricultural labor share (80%), along with higher transport costs (15 yuan) and longer travel time (20 minutes). For broader context, the average income in Qingshuihe County (24,110 yuan) is notably higher than that of the study villages, indicating economic disparities between rural and county-level populations. Moreover, the county’s overall road density (0.65 km/km²) is lower than that of all study villages, as much of the county consists of non-residential land such as mountains, forests, and agricultural fields. This suggests that while rural areas face infrastructural challenges, localized road investments, including the SHRR program, may have enhanced connectivity in specific villages.

| Table 1 | | | | | | | |
| --- | --- | --- | --- | --- | --- | --- | --- |
| Village characteristics. | | | | | | | |
|  | Village A | Village B | Village C | Village D | Qingshuihe County | |  |
| Village Area (km²) | 20 | 35 | 8.7 | 35 | | 2818 |  |
| Road Density (km/km²) | 1.15 | 0.71 | 2.3 | 0.71 | | 0.65 |  |
| Paved Road Percentage (%) | 85 | 93 | 90 | 85 | | N/A |  |
| Average Transport Cost (Yuan) | 10 | 15 | 5 | 15 | | N/A |  |
| SHRR | Yes | No | No | Yes | | N/A |  |
| Road Upgrades in Last 5 Years (km) | 2 | 16 | 2 | 10 | | 781 |  |
| Avg Travel Time to County Center (min) | 15 | 25 | 5 | 20 | | N/A |  |
| Average income (Yuan) | 14500 | 16500 | 15000 | 17000 | | 24110 |  |
| Population (People) | 434 | 998 | 1285 | 1742 | | 136400 |  |
| Ratio of Labor Force Engaged in Agriculture (%) | 81 | 80 | 65 | 80 | | N/A |  |

Source: Survey data and the Statistical Yearbook.

These additions strengthen the economic background of our sample and its relevance to transportation decisions.

**d. Please provide statical information in the table to support “Education data reveal that 37.01% of respondents….primary school education.”**

**Response:** We have updated Table 2 (Table 3) to explicitly include the education distribution percentages for each level (e.g., primary school, middle school, high school, etc.). Additionally, the revised text in Section 4.1 directly refers to these statistics, ensuring full alignment with the data presented in the table (Line:394-400).

| Table 3 | | | | | |  |
| --- | --- | --- | --- | --- | --- | --- |
| Descriptive statistics. | | | | | |  |
| Variables | Obs. | Mean / Percentage Share | S.D. | Min | Max | |
| TM_3 | 127 | 2.188976 | 0.8795046 | 1 | 3 | |
| Car_owners~p | 127 | 0.9685039 | 0.5900902 | 0 | 2 | |
| Average_we~t | 127 | 9.787402 | 4.903238 | 1 | 30 | |
| Average_tr~e | 127 | 2.80315 | 1.291236 | 1 | 5 | |
| SHRR | 127 | 0.5590551 | 0.4984666 | 0 | 1 | |
| Age | 127 | 55.7874 | 8.438978 | 30 | 76 | |
| Incomelev~22 | 127 | 2.787402 | 1.051302 | 1 | 9 | |
| Agri_work_~e | 127 | 0.6232283 | 0.2775771 | 0.1 | 1 | |
| Self_health | 127 | 1.850394 | 0.7671711 | 1 | 5 | |
| Education | 127 | 3.149606 | 1.241343 | 2 | 7 | |
| Primary school | 44 | 34.65 |  |  |  | |
| Middle school | 47 | 37.01 |  |  |  | |
| High school | 24 | 18.9 |  |  |  | |
| Junior college | 9 | 7.09 |  |  |  | |
| University | 3 | 2.36 |  |  |  | |
| BK | 125 | 1.72 | 0.8576036 | 1 | 4 | |
| Improvement in transportation facilities | | |  |  |  | |
|  | 57 | 45.6 |  |  |  | |
| Upgrade or purchase of vehicles | | |  |  |  | |
|  | 57 | 45.6 |  |  |  | |
| Changes in economic conditions | | | | | | |
|  | 11 | 8.8 |  |  |  | |

*Education* data reveal that 37.01% of household heads have completed middle school, and 34.65% have a primary school education. Finally, the *BK* variable (Primary Factor Affecting Travel) shows that 45.60% of household heads identified improvement in transportation facilities as the main factor, while another 45.60% cited the upgrade or purchase of vehicles. A smaller portion, 8.80%, indicated relocation of home or workplace as the primary factor.

**e. “Average” and “mean” are mixed among the table and main texts. Please ensure consistency.**

**Response:** We have standardized the terminology throughout the manuscript, ensuring that “mean” is consistently used in all statistical contexts. The term “average” has been replaced with “mean” wherever applicable, particularly in Tables 3 and their corresponding discussions in Section 4.1. (Line:382-400)

| Table 3 | | | | | |  |
| --- | --- | --- | --- | --- | --- | --- |
| Descriptive statistics. | | | | | |  |
| Variables | Obs. | Mean / Percentage Share | S.D. | Min | Max | |
| TM_3 | 127 | 2.188976 | 0.8795046 | 1 | 3 | |
| Car_owners~p | 127 | 0.9685039 | 0.5900902 | 0 | 2 | |
| Average_we~t | 127 | 9.787402 | 4.903238 | 1 | 30 | |
| Average_tr~e | 127 | 2.80315 | 1.291236 | 1 | 5 | |
| SHRR | 127 | 0.5590551 | 0.4984666 | 0 | 1 | |
| Age | 127 | 55.7874 | 8.438978 | 30 | 76 | |
| Incomelev~22 | 127 | 2.787402 | 1.051302 | 1 | 9 | |
| Agri_work_~e | 127 | 0.6232283 | 0.2775771 | 0.1 | 1 | |
| Self_health | 127 | 1.850394 | 0.7671711 | 1 | 5 | |
| Education | 127 | 3.149606 | 1.241343 | 2 | 7 | |
| Primary school | 44 | 34.65 |  |  |  | |
| Middle school | 47 | 37.01 |  |  |  | |
| High school | 24 | 18.9 |  |  |  | |
| Junior college | 9 | 7.09 |  |  |  | |
| University | 3 | 2.36 |  |  |  | |
| BK | 125 | 1.72 | 0.8576036 | 1 | 4 | |
| Improvement in transportation facilities | | |  |  |  | |
|  | 57 | 45.6 |  |  |  | |
| Upgrade or purchase of vehicles | | |  |  |  | |
|  | 57 | 45.6 |  |  |  | |
| Changes in economic conditions | | | | | | |
|  | 11 | 8.8 |  |  |  | |

The mean of *Average_week_times_goout* is 9.79, reflecting a range from 1 to 30 weekly outings and suggesting varied mobility patterns. *Average_travel_distance* has a mean of 2.803, indicating that most trips fall within the 6-10 km range. The majority of respondents (32.28%) travel between 11-20 km, followed by those who travel less than 1 km (25.98%) and 6-10 km (25.20%). Only 5.51% of respondents travel more than 20 km. The *SHRR* variable reveals that slightly more than half of the villages (mean = 0.559) have implemented SHRR projects, with a relatively balanced distribution across the sample.

The household heads' mean *Age* is 55.79 years, with most being middle-aged or older, and there is moderate variation in income levels (*Incomelevel_22* mean = 2.787), suggesting that the sample is primarily composed of low- to middle-income households. *Agri_work_share* indicates a significant reliance on agriculture, with a mean of 0.623, though this dependence varies across households. Meanwhile, the mean of *Self_health* is 1.850, suggesting generally good health among respondents. *Education* data reveal that 37.01% of household heads have completed middle school, and 34.65% have a primary school education. Finally, the *BK* variable (Primary Factor Affecting Travel) shows that 45.60% of household heads identified improvement in transportation facilities as the main factor, while another 45.60% cited the upgrade or purchase of vehicles. A smaller portion, 8.80%, indicated relocation of home or workplace as the primary factor.

**f. Please describe the average market prices of major transport means in the country and, if data is available, each village.**

**Response:** We appreciate the reviewer’s suggestion and have added a discussion on the average market prices of major transport means in the study villages, using Average Transport Cost (Yuan) as a reference point for travel expenses. Since direct pricing data for specific transport modes in each village is limited, the Average Transport Cost—measured by the average cost to travel to the county center—serves as a proxy for understanding transportation expenses and affordability in Section 3.3. (Line:303-321)

Table 1 presents key characteristics for the four study villages. Village A shows a moderate road density (1.15 km/km²), lower average income (14,500 yuan), and high reliance on agriculture (81%), suggesting a stronger dependence on traditional, local transport modes. Village B, which did not implement the SHRR, has a low road density (0.71 km/km²) but a high paved road percentage (93%) and an average income of 16,500 yuan—indicating that prior infrastructure investments have improved road quality, even if internal connectivity remains limited. In contrast, Village C stands out with the highest road density (2.3 km/km²), the lowest transport cost (5 yuan) and travel time (5 minutes) to the county center, an average income of 15,000 yuan, and a lower agricultural labor share (65%), reflecting superior connectivity and accessibility. Village D, which implemented the SHRR, shares a low road density (0.71 km/km²) with Village B but has the highest average income (17,000 yuan) and a moderate agricultural labor share (80%), along with higher transport costs (15 yuan) and longer travel time (20 minutes). For broader context, the average income in Qingshuihe County (24,110 yuan) is notably higher than that of the study villages, indicating economic disparities between rural and county-level populations. Moreover, the county’s overall road density (0.65 km/km²) is lower than that of all study villages, as much of the county consists of non-residential land such as mountains, forests, and agricultural fields. This suggests that while rural areas face infrastructural challenges, localized road investments, including the SHRR program, may have enhanced connectivity in specific villages.

| Table 1 | | | | | | | |
| --- | --- | --- | --- | --- | --- | --- | --- |
| Village characteristics. | | | | | | | |
|  | Village A | Village B | Village C | Village D | Qingshuihe County | |  |
| Village Area (km²) | 20 | 35 | 8.7 | 35 | | 2818 |  |
| Road Density (km/km²) | 1.15 | 0.71 | 2.3 | 0.71 | | 0.65 |  |
| Paved Road Percentage (%) | 85 | 93 | 90 | 85 | | N/A |  |
| Average Transport Cost (Yuan) | 10 | 15 | 5 | 15 | | N/A |  |
| SHRR | Yes | No | No | Yes | | N/A |  |
| Road Upgrades in Last 5 Years (km) | 2 | 16 | 2 | 10 | | 781 |  |
| Avg Travel Time to County Center (min) | 15 | 25 | 5 | 20 | | N/A |  |
| Average income (Yuan) | 14500 | 16500 | 15000 | 17000 | | 24110 |  |
| Population (People) | 434 | 998 | 1285 | 1742 | | 136400 |  |
| Ratio of Labor Force Engaged in Agriculture (%) | 81 | 80 | 65 | 80 | | N/A |  |

Source: Survey data and the Statistical Yearbook.

This addition helps contextualize affordability concerns and supports our findings on transport mode selection.

**g. How did you consider villages' influences? You may include a dummy variable representing each village at modelling exercises. Furthermore, the descriptive statistics in Table 2 can be divided for each village, which should be possible as the total sample number is 127.**

**Response:** We appreciate the reviewer’s suggestion regarding the inclusion of village-level influences in our analysis. To address this concern, we introduced Section 4.5: Robustness Check to validate our findings by integrating village-level fixed effects into the model estimation. This additional analysis helps isolate the impact of the SHRR program by controlling for unmeasured village-specific characteristics that may influence car ownership, travel behavior, and mode choice. (Line:565-597)

**4.5. Robustness check**

The primary analysis identified a significant relationship between the SHRR program and travel behavior. However, the interpretation of these results could be influenced by unobserved heterogeneity across villages. Since the study area consists of four distinct villages, each with unique socio-economic and infrastructural characteristics, there is a possibility that village-specific factors—such as local economic conditions, alternative transportation availability, land use patterns, or cultural attitudes toward mobility—may affect travel behavior independently of SHRR implementation.

| **Table 6** | | | | | | | | | |
| --- | --- | --- | --- | --- | --- | --- | --- | --- | --- |
| Standardized direct, indirect and total effects on car ownership, travel frequency and travel distance with village fixed effect. | | | | | | | | | |
| Variables | Car_ownership | Average_week_times_goout | | | Average_travel_distance | | |  |  |
|  | Direct | Direct | Indirect | Total | Direct | Indirect | Total | |  |
|  | effect | effect | effect | effect | effect | effect | effect | |  |
| Car_ownership |  | -0.863 |  | -0.863 | 0.516** |  | 0.516** | |  |
|  |  | (-1.19) |  | (-1.19) | (2.90) |  | (2.90) | |  |
| Age | -0.013* | -0.015 | 0.011 | 0.004 | 0.009 | -0.007 | -0.002 | |  |
|  | (-2.05) | (-0.29) | (1.03) | (-0.08) | (0.72) | (-1.67) | (0.20) | |  |
| Education | -0.045 | 0.44 | 0.039 | 0.479 | -0.088 | -0.023 | -0.111 | |  |
|  | (-0.92) | (1.11) | (0.73) | (1.21) | (-0.90) | (-0.88) | (-1.11) | |  |
| Self_health | -0.121 | 0.41 | 0.105 | 0.514 | -0.021 | -0.062 | -0.084 | |  |
|  | (-1.62) | (0.67) | (0.96) | (0.85) | (-0.14) | (-1.41) | (-0.55) | |  |
| Agri_work_share | -0.086 | 0.858 | 0.074 | 0.933 | -0.108 | -0.044 | -0.153 | |  |
|  | (-0.41) | (0.50) | (0.38 | (0.54) | (-0.26) | (-0.40) | (-0.35) | |  |
| Incomelevel_22 | -0.12* | 0.207 | 0.104 | 0.311 | -0.007 | -0.062 | -0.069 | |  |
|  | (-2.40) | (0.50) | (1.07) | (0.77) | (-0.07) | (-1.85) | (-0.67) | |  |
| BK | -0.032 | -0.071 | 0.027 | -0.044 | 0.098 | -0.016 | 0.082 | |  |
|  | (-0.52) | (-0.15) | (0.48) | (-0.09) | (0.82) | (-0.51) | (0.66) | |  |
| SHRR | -0.37* | -4.638** | 0.3196 | -4.319** | 1.202*** | -0.191 | 1.011** | |  |
|  | (-2.09) | (-3.18) | (1.04) | (-2.99) | (3.35) | (-1.69) | (2.77) | |  |
| Village fixed effect | YES | YES | YES | YES | YES | YES |  | |  |
| *R^2^* | 0.127 | 0.201 |  |  | 0.291 |  |  | |  |

Note: 1) *t* statistics in parentheses 2) * p<0.05, ** p<0.01, *** p<0.001

To address these concerns, we re-estimated our models by introducing village fixed effects, using a dummy variable approach where three villages were included as categorical controls, with one village serving as the reference category. The results, presented in Table 6 and 7, confirm the robustness of our initial findings while highlighting some variations in the magnitude of estimated effects.

Table 6 presents the standardized direct, indirect, and total effects of key variables on car ownership, travel frequency, and travel distance while controlling for village fixed effects. The results remain consistent with our initial estimation, reinforcing the negative impact of SHRR on car ownership (direct effect = -0.37, p < 0.05). The coefficient for SHRR on travel frequency remains strongly negative (direct effect =-4.638, p < 0.01; total effect == -4.319, p < 0.01) and exhibits a significant positive effect on travel distance (direct effect =1.202, p < 0.001; total effect = 1.011, p < 0.01). The inclusion of village fixed effects does not alter our finding.

| **Table 7** | | | | | | | |
| --- | --- | --- | --- | --- | --- | --- | --- |
| Integrated SEM and DCM model estimation for standardized direct, indirect and total effects on travel mode choice with village fixed effect. | | | | | | | |
| Variables | Electric bicycle | | | Motorized mode | |  |  |
|  | Direct | Indirect | Total | Direct | Indirect | Total |  |
|  | effect | Effect | effect | effect | effect | effect |  |
| Car_ownership | 1.46 | -0.231 | 1.229 | 2.235*** | -0.131 | 2.103*** |  |
|  | (1.84) | (-0.94) | (1.59) | (3.40) | (-0.63) | (3.35) |  |
| SHRR | 4.298** | -0.682 | 3.616** | 3.252** | -0.685 | 2.567* |  |
|  | (3.05) | (-1.21) | (2.72) | (2.80) | (-1.27) | (2.31) |  |
| Average_travel_distance | -0.41 |  | -0.41 | -0.19 |  | -0.19 |  |
|  | (-1.16) |  | (-1.16) | (-0.61) |  | (-0.61) |  |
| Average_week_times_goout | 0.006 |  | 0.006 | 0.029 |  | 0.029 |  |
|  | (0.07) |  | (0.07) | (0.38) |  | (0.38) |  |
| Household characteristics | Yes | Yes | Yes | Yes | Yes | Yes |  |
| Village fixed effect | Yes | Yes | Yes | Yes | Yes | Yes |  |
| LL_K_/LRI | -745.5922/0.8245 | | | | | |  |
| AIC/Adjust BIC | 267.687/276.22 | | | | | |  |

Note: 1) *t* statistics in parentheses 2) * p<0.05, ** p<0.01, *** p<0.001

Table 7 presents the results from the integrated SEM and DCM estimation of travel mode choice with village fixed effects. The key relationships observed in the initial model remain robust, confirming that SHRR exerts a significant positive effect on electric bicycle adoption (direct effect = 4.298, p < 0.01; total effect = 3.616, p < 0.01). For motorized modes, SHRR also demonstrates a strong direct effect (3.252, p < 0.01) and total effect (2.567, p < 0.05), reinforcing the argument that enhanced rural roads encourage the use of electric bicycle and motorized mode. The shift in statistical significance for the total effect of SHRR on motorized mode adoption highlights the necessity of accounting for village-specific characteristics when analyzing rural mobility patterns. In summary, the robustness check with village fixed effects confirms that SHRR significantly influences travel mode choices, promoting both electric bicycle and motorized mode adoption.

**- 4.2. It is interesting that the rural road policy would negatively impact people’s car ownership. However, the interpretation of modeling results should be carefully made. Please include the discussion of potential external factors, such as prices or market conditions of cars, development of public transportation or shared transportation services, and so on. The discussion is needed to support your finding (within the studied rural context, higher-income households might have better access to alternative modes of transportation, or they might reside in areas where car ownership is less necessary due to better connectivity or availability of services) and need for complementary strategies as described in the last paragraph in this section. Also, I observe a potential contradiction with the implication in Section 4.3 (Car ownership further amplifies this effect by providing the means to travel further). Please check internal consistency.**

**Response:** Thank you for your suggestions. To strengthen the discussion on the negative impact of the SHRR program on car ownership, we have made the following revisions in Section 4.2.
(Line: 419-438)

This result suggests that improvements in rural road infrastructure reduce the need for car ownership, possibly by improving accessibility to alternative transportation modes, including public transit, shared mobility services, and non-motorized options. When road networks are upgraded, travel conditions for buses, ride-sharing services, and other public transport options improve, making them more efficient, reliable, and attractive alternatives to private car use. Additionally, better roads often attract investment in logistics, e-commerce delivery, and mobility-as-a-service (MaaS) solutions, providing further alternatives to car ownership. *Age* also shows a significant negative relationship with car ownership (coefficient = -0.0116, p < 0.05), implying that older individuals are less likely to own cars. This could reflect a decline in mobility needs or driving ability with age, or possibly greater dependence on alternative transport modes among older populations. *Incomelevel_22* has a significant negative effect on car ownership (coefficient = -0.1208, p < 0.05), contrary to the expectation that higher income might be associated with higher car ownership. This finding could suggest that within the studied rural context, higher-income households may be more willing and able to bear the cost of public transportation, reducing their need for private vehicle ownership. Although the four study villages share similar public transport systems, higher-income individuals may utilize public transportation more frequently by prioritizing comfort, reliability, and cost-effectiveness over the long-term expenses associated with car ownership, such as fuel, insurance, maintenance, and depreciation.

We also have delated the contradiction parts in Section 4.3. (Line: 474-475)

These revisions ensure internal consistency and a more robust interpretation of the findings.

**- 4.4. Will you add the roles of electric bicycles in people’s lives in the country and people’s motivations to buy them? Electric bicycles are focused too much in this section, but you may have some ideas to justify this. One reason for the potential unclarity of this section is the absence of each travel mode’s role in people’s lives in the country, and another reason is that “motorized mode” is not clearly defined in the paper’s contexts (sometimes. I read that “motorized mode” means only car, but not always). Please give an in-depth analysis of each travel mode. This ambiguity makes the last paragraph difficult to read and the conclusion statement in Section 5.**

**Response:** To address these concerns, we have made substantial revisions to Section 4.4 and Section 5:

Clarified the definition of “motorized modes” and “electric bicycles”, explicitly differentiating between cars, motorcycles, and electric bicycles to eliminate ambiguity.

Line:485-505

Electric bicycles play a crucial role in rural mobility by offering a cost-effective, flexible, and accessible transportation option. Their affordability and low operational costs make them an attractive alternative to private cars and motorcycles, particularly amid rising fuel prices and maintenance expenses (Cherry & Cervero, 2007; Gu et al., 2021). Electric bicycles provide greater convenience than public transportation, allowing users to travel on their own schedules without reliance on infrequent rural bus services. Additionally, their ease of use—requiring no formal licensing in many areas—enhances accessibility for diverse demographic groups, including women, the elderly, and younger individuals (Weinert et al., 2008). These factors collectively highlight the growing significance of electric bicycles in rural transportation systems and the necessity of integrating infrastructure development with policies that support sustainable and affordable mobility solutions.

Motorized modes, including private cars and motorcycles, remain an essential component of rural travel behavior. Private cars provide higher levels of comfort, safety, and long-distance mobility, making them the preferred option for wealthier households (Zhao & Bai, 2019). However, high costs related to fuel, maintenance, and insurance, coupled with improvements in public transportation and alternative mobility options, may deter car ownership, particularly among lower-income households. Meanwhile, motorcycles continue to serve as a dominant transport mode in rural areas due to their affordability, speed, and ability to navigate unpaved roads. Despite their convenience, motorcycles pose greater safety risks and contribute to higher accident rates compared to other travel modes (Chang et al., 2019). Additionally, government regulations in some regions restricting motorcycle use for safety and environmental reasons have accelerated the shift toward electric bicycles and other alternative transport modes (Gu et al., 2021).

Line:550-564

Car ownership and the SHRR program both show significant positive effects on travel mode choice, suggesting that while these factors enhance overall mobility, they also introduce complexities in the adoption of different transportation modes. Car ownership is strongly associated with an increased likelihood of using motorized modes such as private cars and motorcycles. However, its effect on electric bicycle use is more nuanced, as improved infrastructure and greater mobility options may either encourage or diminish electric bicycle adoption, depending on factors such as travel distance, cost considerations, and individual preferences. Additionally, the significant impact of weekly travel frequency underscores the importance of understanding how daily mobility needs influence transportation choices. Frequent travelers are likely to prioritize modes of transport that can reduce travel time and provide reliable access to various destinations, particularly in rural areas where distances between locations can be substantial.

Refined the concluding statements in Section 5, ensuring consistency in how transport modes interact with infrastructure improvements. Line:608-633

The findings indicate that the SHRR program has a multifaceted impact on travel behavior. Specifically, the program significantly reduces travel frequency, a result likely attributable to improved local accessibility, which decreases the necessity for frequent trips. This suggests that as infrastructure enhances, residents may be able to accomplish more within a single trip, reducing the need for multiple journeys. Concurrently, the SHRR program facilitates longer travel distances and increases reliance on both motorized modes and electric bicycles, reflecting the broader mobility shifts enabled by rural road enhancements. The improved road infrastructure not only makes longer trips more feasible but also encourages the adoption of private cars, motorcycles, and electric bicycles, as these modes offer greater flexibility and efficiency for covering extended distances. Car ownership emerges as a pivotal factor in this context, showing strong positive effects on both travel distance and the likelihood of choosing motorized modes. Households with access to these transport options are better equipped to take advantage of the improved infrastructure, resulting in more frequent and longer trips. However, the relationship between car ownership and the use of electric bicycles is more complex. While infrastructure and car ownership collectively enhance mobility, they may simultaneously reduce the necessity or appeal of certain transport modes, particularly electric bicycles, depending on trip purpose, cost considerations, and individual travel preferences. These findings highlight the dual impact of infrastructure improvements: while fostering greater mobility and travel efficiency, they also reshape transport mode preferences. This study contributes to the understanding of rural mobility dynamics by providing empirical evidence on how infrastructure interventions influence travel behaviors, emphasizing the need for integrated policy approaches that account for both direct and indirect effects on transport choices.

These modifications provide a more comprehensive and structured discussion of transportation choices in rural contexts.

We added following references accordingly:

Chang, F., Xu, P., Zhou, H., Lee, J., & Huang, H. (2019). Identifying motorcycle high-risk traffic scenarios through interactive analysis of driver behavior and traffic characteristics. *Transportation research part F: traffic psychology and behaviour, 62*, 844-854.

Cherry, C., & Cervero, R. (2007). Use characteristics and mode choice behavior of electric bike users in China. *Transport policy, 14*(3), 247-257.

Gu, T., Kim, I., & Currie, G. (2021). The two-wheeled renaissance in China—An empirical review of bicycle, E-bike, and motorbike development. *International journal of sustainable transportation, 15*(4), 239-258.

MOT. (2015). *Opinions of the Ministry of Transport on Promoting the Construction of “Sihao Rural Roads" (in Chinese)*. Retrieved October 9th from <https://www.mot.gov.cn/zhengcejiedu/sihaoncl/xiangguanzhengce/201808/t20180810_3056482.html>

MOT. (2019). *Guiding Opinions on Promoting the High-Quality Development of “Sihao Rural Roads" (in Chinese)*. Retrieved October 9th from <https://www.mot.gov.cn/zhengcejiedu/tdshncl/>

Weinert, J., Ogden, J., Sperling, D., & Burke, A. (2008). The future of electric two-wheelers and electric vehicles in China. *Energy Policy, 36*(7), 2544-2555.

Zhao, P., & Bai, Y. (2019). The gap between and determinants of growth in car ownership in urban and rural areas of China: A longitudinal data case study. *Journal of Transport Geography, 79*, 102487.

**Reviewer #2: In comparing the studies in the four villages, it is undeniable that the differences in the transportation behavior of the people in these villages are due to conditions other than the presence or absence of the SHRR Policy.The SEM and the Descrete choice model framework and its thin description of the results do not allow us to judge the validity of this model and results. Overall, the simplicity of the survey makes it difficult to conclude the impact of the SHRR Policy from this alone.**

**Response:** Thank you for your detailed comments. We appreciate your concern regarding potential confounding influences in our comparative analysis, the clarity of our modeling framework, and the simplicity of our survey design. Below, we address each point in turn:

1. Accounting for Village-Level Differences

We recognize that differences in transportation behavior across villages may be influenced by factors beyond the presence or absence of the Sihao Rural Road (SHRR) policy. To address this, we have incorporated village fixed effects into our Structural Equation Model (SEM) and Discrete Choice Model (DCM) estimation to account for unobserved heterogeneity. The revised results confirm the robustness of our findings, with the inclusion of village-level controls strengthening the statistical significance of key relationships (Table 6 and Table 7). Additionally, a descriptive breakdown of socio-economic characteristics by village (Table 1) has been added to better contextualize differences in income, road density, and transport costs.

(Line:303-321)

Table 1 presents key characteristics for the four study villages. Village A shows a moderate road density (1.15 km/km²), lower average income (14,500 yuan), and high reliance on agriculture (81%), suggesting a stronger dependence on traditional, local transport modes. Village B, which did not implement the SHRR, has a low road density (0.71 km/km²) but a high paved road percentage (93%) and an average income of 16,500 yuan—indicating that prior infrastructure investments have improved road quality, even if internal connectivity remains limited. In contrast, Village C stands out with the highest road density (2.3 km/km²), the lowest transport cost (5 yuan) and travel time (5 minutes) to the county center, an average income of 15,000 yuan, and a lower agricultural labor share (65%), reflecting superior connectivity and accessibility. Village D, which implemented the SHRR, shares a low road density (0.71 km/km²) with Village B but has the highest average income (17,000 yuan) and a moderate agricultural labor share (80%), along with higher transport costs (15 yuan) and longer travel time (20 minutes). For broader context, the average income in Qingshuihe County (24,110 yuan) is notably higher than that of the study villages, indicating economic disparities between rural and county-level populations. Moreover, the county’s overall road density (0.65 km/km²) is lower than that of all study villages, as much of the county consists of non-residential land such as mountains, forests, and agricultural fields. This suggests that while rural areas face infrastructural challenges, localized road investments, including the SHRR program, may have enhanced connectivity in specific villages.

| Table 1 | | | | | | | |
| --- | --- | --- | --- | --- | --- | --- | --- |
| Village characteristics. | | | | | | | |
|  | Village A | Village B | Village C | Village D | Qingshuihe County | |  |
| Village Area (km²) | 20 | 35 | 8.7 | 35 | | 2818 |  |
| Road Density (km/km²) | 1.15 | 0.71 | 2.3 | 0.71 | | 0.65 |  |
| Paved Road Percentage (%) | 85 | 93 | 90 | 85 | | N/A |  |
| Average Transport Cost (Yuan) | 10 | 15 | 5 | 15 | | N/A |  |
| SHRR | Yes | No | No | Yes | | N/A |  |
| Road Upgrades in Last 5 Years (km) | 2 | 16 | 2 | 10 | | 781 |  |
| Avg Travel Time to County Center (min) | 15 | 25 | 5 | 20 | | N/A |  |
| Average income (Yuan) | 14500 | 16500 | 15000 | 17000 | | 24110 |  |
| Population (People) | 434 | 998 | 1285 | 1742 | | 136400 |  |
| Ratio of Labor Force Engaged in Agriculture (%) | 81 | 80 | 65 | 80 | | N/A |  |

Source: Survey data and the Statistical Yearbook.

Line:565-597

**4.5. Robustness check**

The primary analysis identified a significant relationship between the SHRR program and travel behavior. However, the interpretation of these results could be influenced by unobserved heterogeneity across villages. Since the study area consists of four distinct villages, each with unique socio-economic and infrastructural characteristics, there is a possibility that village-specific factors—such as local economic conditions, alternative transportation availability, land use patterns, or cultural attitudes toward mobility—may affect travel behavior independently of SHRR implementation.

To address these concerns, we re-estimated our models by introducing village fixed effects, using a dummy variable approach where three villages were included as categorical controls, with one village serving as the reference category. The results, presented in Table 6 and 7, confirm the robustness of our initial findings while highlighting some variations in the magnitude of estimated effects.

Table 6 presents the standardized direct, indirect, and total effects of key variables on car ownership, travel frequency, and travel distance while controlling for village fixed effects. The results remain consistent with our initial estimation, reinforcing the negative impact of SHRR on car ownership (direct effect = -0.37, p < 0.05). The coefficient for SHRR on travel frequency remains strongly negative (direct effect =-4.638, p < 0.01; total effect == -4.319, p < 0.01) and exhibits a significant positive effect on travel distance (direct effect =1.202, p < 0.001; total effect = 1.011, p < 0.01). The inclusion of village fixed effects does not alter our finding.

Table 7 presents the results from the integrated SEM and DCM estimation of travel mode choice with village fixed effects. The key relationships observed in the initial model remain robust, confirming that SHRR exerts a significant positive effect on electric bicycle adoption (direct effect = 4.298, p < 0.01; total effect = 3.616, p < 0.01). For motorized modes, SHRR also demonstrates a strong direct effect (3.252, p < 0.01) and total effect (2.567, p < 0.05), reinforcing the argument that enhanced rural roads encourage the use of electric bicycle and motorized mode. The shift in statistical significance for the total effect of SHRR on motorized mode adoption highlights the necessity of accounting for village-specific characteristics when analyzing rural mobility patterns. In summary, the robustness check with village fixed effects confirms that SHRR significantly influences travel mode choices, promoting both electric bicycle and motorized mode adoption.

| **Table 6** | | | | | | | | | |
| --- | --- | --- | --- | --- | --- | --- | --- | --- | --- |
| Standardized direct, indirect and total effects on car ownership, travel frequency and travel distance with village fixed effect. | | | | | | | | | |
| Variables | Car_ownership | Average_week_times_goout | | | Average_travel_distance | | |  |  |
|  | Direct | Direct | Indirect | Total | Direct | Indirect | Total | |  |
|  | effect | effect | effect | effect | effect | effect | effect | |  |
| Car_ownership |  | -0.863 |  | -0.863 | 0.516** |  | 0.516** | |  |
|  |  | (-1.19) |  | (-1.19) | (2.90) |  | (2.90) | |  |
| Age | -0.013* | -0.015 | 0.011 | 0.004 | 0.009 | -0.007 | -0.002 | |  |
|  | (-2.05) | (-0.29) | (1.03) | (-0.08) | (0.72) | (-1.67) | (0.20) | |  |
| Education | -0.045 | 0.44 | 0.039 | 0.479 | -0.088 | -0.023 | -0.111 | |  |
|  | (-0.92) | (1.11) | (0.73) | (1.21) | (-0.90) | (-0.88) | (-1.11) | |  |
| Self_health | -0.121 | 0.41 | 0.105 | 0.514 | -0.021 | -0.062 | -0.084 | |  |
|  | (-1.62) | (0.67) | (0.96) | (0.85) | (-0.14) | (-1.41) | (-0.55) | |  |
| Agri_work_share | -0.086 | 0.858 | 0.074 | 0.933 | -0.108 | -0.044 | -0.153 | |  |
|  | (-0.41) | (0.50) | (0.38 | (0.54) | (-0.26) | (-0.40) | (-0.35) | |  |
| Incomelevel_22 | -0.12* | 0.207 | 0.104 | 0.311 | -0.007 | -0.062 | -0.069 | |  |
|  | (-2.40) | (0.50) | (1.07) | (0.77) | (-0.07) | (-1.85) | (-0.67) | |  |
| BK | -0.032 | -0.071 | 0.027 | -0.044 | 0.098 | -0.016 | 0.082 | |  |
|  | (-0.52) | (-0.15) | (0.48) | (-0.09) | (0.82) | (-0.51) | (0.66) | |  |
| SHRR | -0.37* | -4.638** | 0.3196 | -4.319** | 1.202*** | -0.191 | 1.011** | |  |
|  | (-2.09) | (-3.18) | (1.04) | (-2.99) | (3.35) | (-1.69) | (2.77) | |  |
| Village fixed effect | YES | YES | YES | YES | YES | YES |  | |  |
| *R^2^* | 0.127 | 0.201 |  |  | 0.291 |  |  | |  |

Note: 1) *t* statistics in parentheses 2) * p<0.05, ** p<0.01, *** p<0.001

| **Table 7** | | | | | | | |
| --- | --- | --- | --- | --- | --- | --- | --- |
| Integrated SEM and DCM model estimation for standardized direct, indirect and total effects on travel mode choice with village fixed effect. | | | | | | | |
| Variables | Electric bicycle | | | Motorized mode | |  |  |
|  | Direct | Indirect | Total | Direct | Indirect | Total |  |
|  | effect | Effect | effect | effect | effect | effect |  |
| Car_ownership | 1.46 | -0.231 | 1.229 | 2.235*** | -0.131 | 2.103*** |  |
|  | (1.84) | (-0.94) | (1.59) | (3.40) | (-0.63) | (3.35) |  |
| SHRR | 4.298** | -0.682 | 3.616** | 3.252** | -0.685 | 2.567* |  |
|  | (3.05) | (-1.21) | (2.72) | (2.80) | (-1.27) | (2.31) |  |
| Average_travel_distance | -0.41 |  | -0.41 | -0.19 |  | -0.19 |  |
|  | (-1.16) |  | (-1.16) | (-0.61) |  | (-0.61) |  |
| Average_week_times_goout | 0.006 |  | 0.006 | 0.029 |  | 0.029 |  |
|  | (0.07) |  | (0.07) | (0.38) |  | (0.38) |  |
| Household characteristics | Yes | Yes | Yes | Yes | Yes | Yes |  |
| Village fixed effect | Yes | Yes | Yes | Yes | Yes | Yes |  |
| LL_K_/LRI | -745.5922/0.8245 | | | | | |  |
| AIC/Adjust BIC | 267.687/276.22 | | | | | |  |

Note: 1) *t* statistics in parentheses 2) * p<0.05, ** p<0.01, *** p<0.001

2. Clarifying the Justification for Using Travel Behavior as an Analytical Focus

We have moved the three key research questions from Section 4.1 to the Introduction (Lines: 121-138) and explicitly clarified the rationale for examining travel behavior as a primary outcome of rural road improvements.

We utilize an integrated Structural Equation Modeling (SEM) and Discrete Choice Modeling (DCM) framework to capture both the direct and indirect effects of the SRRP on travel behavior, thus offering a more nuanced understanding of the mechanisms behind these changes.

In particular, the research addresses the following questions:

(1) Does the SRRP continue to exert significant direct effects on car ownership, travel frequency, travel distance, and travel mode choice after controlling for socio-demographic factors?
(2) How does the SRRP indirectly influence travel mode choice through its effects on mediating variables such as car ownership, travel frequency, and travel distance?
(3) What is the overall impact of the SRRP on travel mode choice, combining both direct and indirect effects?

By examining these questions, the study aims to provide a comprehensive perspective on how rural infrastructure improvements like the SRRP shape travel behavior. Findings from this research can inform policy recommendations for future rural road development and identify ways to optimize infrastructure investments to achieve broader socio-economic benefits and sustainable mobility patterns.

Additionally, we have explicitly clarified why measuring the impact of the Sihao Rural Road (SHRR) program necessitates a focus on travel behaviors. (Line:88-115)

The decision to focus on travel behavior stems from the critical role that daily mobility plays in realizing the broader benefits of rural road improvements. Enhanced rural roads can fundamentally reshape how often residents travel, how far they go, and which modes of transport they prefer. These changes in travel behavior mediate a wide range of socioeconomic outcomes: improved roads may reduce the time and financial costs of commuting, prompt shifts in mode preference (e.g., from walking to motorized modes), and influence household decisions about vehicle ownership. By capturing these direct and indirect pathways, researchers gain a more comprehensive understanding of how the SRRP translates into real-world changes in mobility patterns—ultimately affecting economic opportunities, access to services, and social inclusion.

The SRRP, initiated in 2015, is particularly interesting because it aims to fix the differences in transport infrastructure between urban and rural areas (MOT, 2015). By focusing on improving the quality and connectivity of rural roads, the policy aspires not only to make travel more convenient but also to foster new economic opportunities in rural areas. This could help close the persistent urban–rural income gap, a central concern for sustainable development. Through enhanced transport links, rural communities could see boosts in employment, market access, and overall well-being (MOT, 2019). Despite the program’s potential, there is still limited empirical evidence demonstrating how specific aspects of the SRRP—like upgraded road infrastructure and enhanced road management—translate into shifts in travel behavior and ultimately contribute to rural revitalization.

3. Addressing Concerns About Model Validity and Interpretation

We recognize the concern that our SEM-DCM framework requires a clearer justification and more detailed reporting of results. In response, we have:

• Expanded the discussion on how SEM captures direct and indirect effects of SHRR on transport mode choice, we have made the following revisions in Section 4.2.
(Line: 419-438)

This result suggests that improvements in rural road infrastructure reduce the need for car ownership, possibly by improving accessibility to alternative transportation modes, including public transit, shared mobility services, and non-motorized options. When road networks are upgraded, travel conditions for buses, ride-sharing services, and other public transport options improve, making them more efficient, reliable, and attractive alternatives to private car use. Additionally, better roads often attract investment in logistics, e-commerce delivery, and mobility-as-a-service (MaaS) solutions, providing further alternatives to car ownership. *Age* also shows a significant negative relationship with car ownership (coefficient = -0.0116, p < 0.05), implying that older individuals are less likely to own cars. This could reflect a decline in mobility needs or driving ability with age, or possibly greater dependence on alternative transport modes among older populations. *Incomelevel_22* has a significant negative effect on car ownership (coefficient = -0.1208, p < 0.05), contrary to the expectation that higher income might be associated with higher car ownership. This finding could suggest that within the studied rural context, higher-income households may be more willing and able to bear the cost of public transportation, reducing their need for private vehicle ownership. Although the four study villages share similar public transport systems, higher-income individuals may utilize public transportation more frequently by prioritizing comfort, reliability, and cost-effectiveness over the long-term expenses associated with car ownership, such as fuel, insurance, maintenance, and depreciation.

• Revised Section 4.4 to clarify the role of electric bicycles and motorized modes in rural travel decisions.

Clarified the definition of “motorized modes” and “electric bicycles”, explicitly differentiating between cars, motorcycles, and electric bicycles to eliminate ambiguity.

Line:485-505

Electric bicycles play a crucial role in rural mobility by offering a cost-effective, flexible, and accessible transportation option. Their affordability and low operational costs make them an attractive alternative to private cars and motorcycles, particularly amid rising fuel prices and maintenance expenses (Cherry & Cervero, 2007; Gu et al., 2021). Electric bicycles provide greater convenience than public transportation, allowing users to travel on their own schedules without reliance on infrequent rural bus services. Additionally, their ease of use—requiring no formal licensing in many areas—enhances accessibility for diverse demographic groups, including women, the elderly, and younger individuals (Weinert et al., 2008). These factors collectively highlight the growing significance of electric bicycles in rural transportation systems and the necessity of integrating infrastructure development with policies that support sustainable and affordable mobility solutions.

Motorized modes, including private cars and motorcycles, remain an essential component of rural travel behavior. Private cars provide higher levels of comfort, safety, and long-distance mobility, making them the preferred option for wealthier households (Zhao & Bai, 2019). However, high costs related to fuel, maintenance, and insurance, coupled with improvements in public transportation and alternative mobility options, may deter car ownership, particularly among lower-income households. Meanwhile, motorcycles continue to serve as a dominant transport mode in rural areas due to their affordability, speed, and ability to navigate unpaved roads. Despite their convenience, motorcycles pose greater safety risks and contribute to higher accident rates compared to other travel modes (Chang et al., 2019). Additionally, government regulations in some regions restricting motorcycle use for safety and environmental reasons have accelerated the shift toward electric bicycles and other alternative transport modes (Gu et al., 2021).

Line:550-564

Car ownership and the SHRR program both show significant positive effects on travel mode choice, suggesting that while these factors enhance overall mobility, they also introduce complexities in the adoption of different transportation modes. Car ownership is strongly associated with an increased likelihood of using motorized modes such as private cars and motorcycles. However, its effect on electric bicycle use is more nuanced, as improved infrastructure and greater mobility options may either encourage or diminish electric bicycle adoption, depending on factors such as travel distance, cost considerations, and individual preferences. Additionally, the significant impact of weekly travel frequency underscores the importance of understanding how daily mobility needs influence transportation choices. Frequent travelers are likely to prioritize modes of transport that can reduce travel time and provide reliable access to various destinations, particularly in rural areas where distances between locations can be substantial.

4. Acknowledging Survey Scope and Sample Size Limitations

We now explicitly discuss the survey methodology, sampling constraints, and data collection challenges in Section 3.3. Our final sample of 127 households, while providing sufficient power for medium-to-large effect sizes, may limit the detection of subtle interactions. We acknowledge this in Section 5 (Lines: 628-632) and recommend that future studies expand the sample size to validate the findings.

We have added the following discussion to Section 3.3 for data collection challenges: (Line:291-296)

We used a stratified sampling technique to select 200 households for the survey, which was distributed across four villages village A, village B, village C, and village D in Qingshuihe County. However, due to incomplete questionnaires (n = 10), data inconsistencies (n = 18), and non-responses (n = 45), a total of 73 responses were excluded. As a result, the final sample for analysis comprised 127 valid responses.

While the final sample of 127 households provided sufficient statistical power to detect medium-to-large effect sizes within the structural equation modeling (SEM) framework, it may have limited the detection of smaller interaction effects. To acknowledge this limitation, we have added a discussion in Section 5, emphasizing the need for future studies with larger and more diverse samples to enhance the robustness of the findings. (Line:634-638)

Despite the valuable insights provided by this study, several limitations should be acknowledged. First, the sample size is relatively small (127 households across four villages), which may limit the generalizability of the findings to other rural areas in China or beyond. While the inclusion of village fixed effects improves robustness, additional research with a larger and more diverse sample could help validate and refine the observed relationships.

5. Ensuring Internal Consistency

We have revised Sections 4.2 and 4.3 to remove potential contradictions in the discussion of car ownership and travel mode choice. We also rewrite the concluding section (Lines: 740-765) to align findings more clearly. (Line:599-652)

5. Conclusion

This study set out to examine the impact of the SHRR Program on travel behavior in rural areas, specifically focusing on how infrastructure improvements influence travel mode choice, travel frequency, and travel distance. Using an integrated SEM and DCM framework, the research analyzed data from 127 households across four villages in Qingshuihe County, Inner Mongolia, China collected between August 2023 and March 2024. The study incorporated key socio-demographic variables, such as car ownership, income levels, education, and self-reported health, to provide a comprehensive understanding of the factors that shape mobility patterns in these rural settings.

The findings indicate that the SHRR program has a multifaceted impact on travel behavior. Specifically, the program significantly reduces travel frequency, a result likely attributable to improved local accessibility, which decreases the necessity for frequent trips. This suggests that as infrastructure enhances, residents may be able to accomplish more within a single trip, reducing the need for multiple journeys. Concurrently, the SHRR program facilitates longer travel distances and increases reliance on both motorized modes and electric bicycles, reflecting the broader mobility shifts enabled by rural road enhancements. The improved road infrastructure not only makes longer trips more feasible but also encourages the adoption of private cars, motorcycles, and electric bicycles, as these modes offer greater flexibility and efficiency for covering extended distances. Car ownership emerges as a pivotal factor in this context, showing strong positive effects on both travel distance and the likelihood of choosing motorized modes. Households with access to these transport options are better equipped to take advantage of the improved infrastructure, resulting in more frequent and longer trips. However, the relationship between car ownership and the use of electric bicycles is more complex. While infrastructure and car ownership collectively enhance mobility, they may simultaneously reduce the necessity or appeal of certain transport modes, particularly electric bicycles, depending on trip purpose, cost considerations, and individual travel preferences. These findings highlight the dual impact of infrastructure improvements: while fostering greater mobility and travel efficiency, they also reshape transport mode preferences. This study contributes to the understanding of rural mobility dynamics by providing empirical evidence on how infrastructure interventions influence travel behaviors, emphasizing the need for integrated policy approaches that account for both direct and indirect effects on transport choices.

Despite the valuable insights provided by this study, several limitations should be acknowledged. First, the sample size is relatively small (127 households across four villages), which may limit the generalizability of the findings to other rural areas in China or beyond. While the inclusion of village fixed effects improves robustness, additional research with a larger and more diverse sample could help validate and refine the observed relationships. Second, although the study employs an integrated SEM and DCM framework to examine the direct and indirect effects of SHRR on travel behavior, potential endogeneity issues remain. Factors such as unobserved individual preferences, self-selection bias, and unmeasured policy interventions could influence transport choices. Future studies could benefit from instrumental variable approaches or longitudinal data to better isolate causal relationships. Third, while this study highlights the role of electric bicycles, private cars, and motorcycles, it does not delve into detailed household decision-making processes regarding transport mode adoption. Future research could explore qualitative factors, such as perceptions of convenience, safety, affordability, and environmental sustainability, to complement quantitative modeling.

We added following references accordingly:

Chang, F., Xu, P., Zhou, H., Lee, J., & Huang, H. (2019). Identifying motorcycle high-risk traffic scenarios through interactive analysis of driver behavior and traffic characteristics. *Transportation research part F: traffic psychology and behaviour, 62*, 844-854.

Cherry, C., & Cervero, R. (2007). Use characteristics and mode choice behavior of electric bike users in China. *Transport policy, 14*(3), 247-257.

Gu, T., Kim, I., & Currie, G. (2021). The two-wheeled renaissance in China—An empirical review of bicycle, E-bike, and motorbike development. *International journal of sustainable transportation, 15*(4), 239-258.

MOT. (2015). *Opinions of the Ministry of Transport on Promoting the Construction of “Sihao Rural Roads" (in Chinese)*. Retrieved October 9th from <https://www.mot.gov.cn/zhengcejiedu/sihaoncl/xiangguanzhengce/201808/t20180810_3056482.html>

MOT. (2019). *Guiding Opinions on Promoting the High-Quality Development of “Sihao Rural Roads" (in Chinese)*. Retrieved October 9th from <https://www.mot.gov.cn/zhengcejiedu/tdshncl/>

Weinert, J., Ogden, J., Sperling, D., & Burke, A. (2008). The future of electric two-wheelers and electric vehicles in China. *Energy Policy, 36*(7), 2544-2555.

Zhao, P., & Bai, Y. (2019). The gap between and determinants of growth in car ownership in urban and rural areas of China: A longitudinal data case study. *Journal of Transport Geography, 79*, 102487.

In sum, we have strengthened the methodological transparency and expanded our discussion of both potential confounders and the validity of the SEM-DCM approach. While we stand by the utility of our analysis in uncovering important insights about how rural road policies shape travel behavior, we also clearly outline its limitations and the need for additional research. We hope these revisions address your concerns and enhance the clarity, rigor, and credibility of our study.

**End**

**February 18, 2025**
